# Antibody Epitope Specificity for dsDNA Phosphate Backbone Is an Intrinsic Property of the Heavy Chain Variable Germline Gene Segment Used

**DOI:** 10.3389/fimmu.2018.02378

**Published:** 2018-10-18

**Authors:** Tatjana Srdic-Rajic, Heinz Kohler, Vladimir Jurisic, Radmila Metlas

**Affiliations:** ^1^Department of Experimental Pharmacology, National Cancer Research Center, Belgrade, Serbia; ^2^Department of Microbiology and Immunology, University of Kentucky, Lexington, KY, United States; ^3^Faculties of Medicinal Science, University of Kragujevac, Kragujevac, Serbia; ^4^Vinča Institute of Nuclear Science, University of Belgrade, Belgrade, Serbia

**Keywords:** anti-DNA antibodies, anti-PC antibodies, VH germline genes, Characterization of antibody specificity by ISM, dsDNA reactive antibodies

## Abstract

Analysis of protein sequences by the informational spectrum method (ISM) enables characterization of their specificity according to encoded information represented with defined frequency (F). Our previous data showed that F(0.367) is characteristic for variable heavy chain (VH) domains (a combination of variable (V), diversity (D) and joining (J) gene segments) of the anti-phosphocholine (PC) T15 antibodies and mostly dependent on the CDR2 region, a site for PC phosphate group binding. Because the T15 dsDNA-reactive U4 mutant also encodes F(0.367), we hypothesized that the same frequency may also be characteristic for anti-DNA antibodies. Data obtained from an analysis of 60 spontaneously produced anti-DNA antibody VH domain sequences supported our hypothesis only for antibodies, which use V gene segment in germline configuration, such as S57(VH31), MRL-DNA22, and VH11, members of the VH1 (J558) and VH7 (S107) gene families. The important finding is that out of seven V gene segments used by spontaneous anti-DNA antibodies, F(0.367) is only expressed by the germline configuration of these three V gene segments. The data suggest that antibody specificity for the phosphate group moiety delineated as F(0.367) is the intrinsic property of the V germline gene segments used, whereas paratope/epitope interaction with antigens bearing this epitope, such as PC or dsDNA, requires corresponding antibody VH conformation that is susceptible to somatic mutation(s).

## Introduction

Natural autoantibodies, mainly IgM whose heavy chains are encoded by unmutated VDJ genes, play a role in immune system homeostasis, provide the first line of defense against infections, and may play a role in autoimmune disease as somatically mutated IgG autoantibodies (1, 2). The highly diverse CDR3 loops are assumed as the key determinant of specificity in antigen recognition, but in nonsomatically mutated antibodies, binding sites may consist of germline-encoded CDR1 and CDR2 sequences dominating in a number of contacts, whereas light chains play a subsidiary role to heavy chains (3, 4). It was also suggested that in contrast to antigen specificity determined by CDR3 (5), germline-encoded CDR1 and CDR2 sequences accommodate binding to a number of different unrelated antigens (6). The analyses also showed that despite the potential to generate almost unlimited variability, the CDR regions exhibit a small number of core main chain conformations termed “canonical structures” (7). In particular, a limited repertoire of the main chain adopted conformations dependent on the loop length and a few key conserved residues at defined positions (8) has been assigned to CDR1 and CDR2 regions (9).

One of the best studied primary antibody responses to phosphocholine (PC) is T15 antibody expressing heavy and light chain products of the T15(V1) and Vk22 germline genes in mice (10–13). It is of interest that in ontogeny, T15 predominant clonotypes appear about 1 week after birth (14), whereas PC-specific responses or precursors were detected as early as 1 day after birth (15). An important finding is that the heavy chains of T15 and other PC binding proteins bearing M603 and M167 idiotypic determinants are derived from a single germline T15(V1) gene segment and three light chains, i.e., T15 (VK22), M603 (VK8), and M167 (VK24) (13, 16, 17).

Crystallography studies of the anti-PC binding antibody provide evidence for the PC contact residues, revealing that favorable interaction of the choline moiety is with CDR1 Glu-35, whereas specific interactions occur between the phosphate group and charged groups such as CDR2 Arg-52 that produce a large favorable electrostatic interaction and Lys-54 that helps neutralize the PC negative charge (18, 19). The data obtained from mutagenesis experiments conferred importance of CDR2 Arg-52 as a site for interaction with the PC phosphate group (20), whereas interaction with the carrier involves different sites (21). The role of CDR2 H52-H56 motif in nucleic acid binding was also demonstrated by analyses of monoclonal autoantibodies derived from lupus-prone mice (22).

On the other hand, T15 CDR2 sequence VH50-60 region, a part of the self-binding domain (homophilicity), enhances antibody potency (23). The CDR2 of T15 antibody, according to our view, may also have an immunoregulatory role in the ontogeny of natural Tregs and consequently in the control of T15 and some anti-DNA antibody diversification (24).

Anti-DNA antibodies recognize a considerable number of different epitopes, and their exact nature is only partially known (25). Anti-dsDNA antibodies may react with linear and conformational determinants exposed on the double helix of DNA and cross-react with different antigens (26). For example, a similar arrangement of phosphate groups in the DNA sugar-phosphate backbone and phospholipids may explain cross-reactivity (27).

Sequence analysis of anti-dsDNA antibodies from autoimmune mice revealed a high frequency of mutations and the presence of basic amino acids in the CDRs, such as Arg and Lys and polar Asn with the potential to interact with structures within dsDNA (28–31) or, when gained during affinity maturation, be critical for CDR3 region interaction with histone-DNA complex (32–34). This complex according to a hapten-carrier-like model, may initiate production of both anti-dsDNA and other anti-nucleosome antibodies [reviewed in (35)].

In prior studies, we have shown that antibody VH domains of anti-PC T15 and T15 dsDNA binding somatic mutant, U4 (13), encode characteristic sequence information represented with F(0.367) (36). In this report, we extended this finding by showing that F(0.367) is also expressed by several anti-DNA antibody VH domains that use V germline or somatically mutated S57(VH31), MRL-DNA22, and VH11 gene segments of the VH1 (J558) and VH7 (S107) gene families, as well as that protein sequences of these germline genes in addition to T15(V1) encode an intrinsic epitope specificity represented by F(0.367). Obtained data suggests that as long as the frequency is expressed by an antibody VH domain (a) the corresponding conformation for paratope/epitope interaction might be preserved despite somatic mutations and (b) because of somatic mutation(s), interaction with another antigen bearing the same epitope might be achieved and vice versa, loss of the characteristic frequency may cause achievement of a new epitope specificity.

## Method

The sequence analysis was performed by applying the informational spectrum method (ISM). The physicomathematical basis of ISM was described in detail elsewhere (37), and here, we will only point the basic steps involved by the method. According to the ISM approach, also denoted as resonant recognition model (RRM) (38), protein sequences are transformed into signals by assignment of numerical values of each amino acid. These values correspond to electron–ion interaction potential (39) determining electronic properties of amino acids that are responsible for their intermolecular interactions (40–43). The signal obtained is decomposed in periodical function by Fourier transformation. The result is a series of frequencies and their amplitudes (the informational spectrum, IS). Detailed steps (43) that precede obtaining the IS by the ISM are explained in the Supplementary Information. The obtained frequencies correspond to the distribution of structural motifs with defined physicochemical characteristics determining the biological function of the sequence. When comparing proteins that share the same biological function, the technique allows detection of code/frequency pairs in IS, which are specific for their common biological properties. This common information is represented by characteristic peaks in the cross-spectrum (CIS) of proteins. The method is insensitive to the location of the motifs and, thus, does not require the previous alignment of the sequence. A measure of similarity for each peak is a signal-to-noise ratio (S/N), which represents a ratio between signal intensity at one particular IS frequency and the mean value of the whole spectrum which depends on the number of the sequences used in the analysis.

## Results

**Figure 1:**
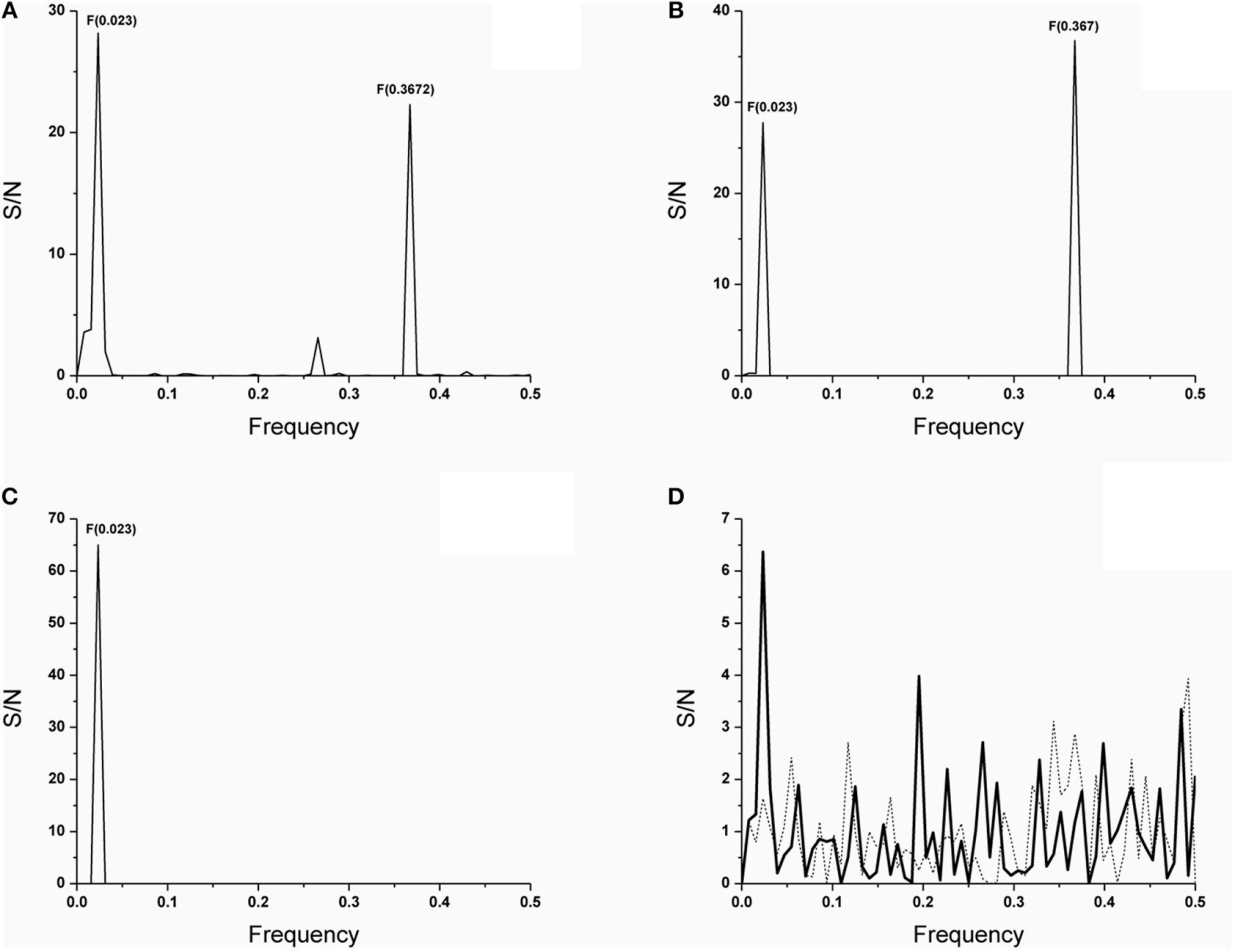
ISM analysis of the V germline genes and antibody VH domain protein sequences. The CIS of the V germline genes segments of VH1, VH11, S57(VH31) and MRL-DNA22 which shows characteristic F(0.367) relevant for the biological activity here followed and activity irrelevant F(0.023) **(A)**. The CIS of the VH domains of antibodies 74.c2 encoded by the V gene segment VH11, a member of the VH7 (S107) gene family, 17s-c2 encoded by the V gene segment S57(VH31) and 17s.83, 17s-c3, 111.185 and 165.27 encoded by V gene segment MRL-DNA22 of the VH1 (J558) gene family **(B)**. CIS of all anti-DNA antibodies which in individual spectrum does not express F(0.367) **(C)**. The IS of the preimmune natural polyreactive autoantibodies which use V gene segments from VH2 (Q52) gene family such as D23 VH domain (___) and superimposed IS of the E7 VH domain (……) **(D)**. The abscissa represents the frequencies from the Fourier transform of the sequence of electron-ion interaction potential (EIIP). The lowest frequency is 0.0 and the highest is 0.5. The ordinate represents the signal to noise ratio (S/N) corresponding to each frequency component in the informational spectrum (IS).

Our previous data showed that VH domain of the anti-PC T15 idiotype antibody that uses an unmutated copy of the V germline gene T15(V1) (16, 17), as well as anti-PC binding antibodies of different idiotypes, encode information represented with F(0.367) in short F(0.37) (36). We also showed that F(0.37), is independent of a single substitution-glutamic acid to alanine, at position 35 in the T15 antibody CDR1 region, causing reactivity acquisition for dsDNA (13) but depends on mutations in CDR2 region (36). In this report, seven V germline gene amino acid sequences used by spontaneous anti-DNA antibodies (31) were analyzed; of which, only three showed F(0.367) in individual spectra such as S57(VH31) (30), MRL-DNA22 (44) germline gene segments members of the VH1(J558) gene family, and VH11 (45) member of the VH7(S107) gene family. The CIS of the T15(V1), S57(VH31), MRL-DNA22, and VH11 V germline gene segment amino acid sequences is presented in Figure 1A, revealing a peak at F(0.367). The T15(V1) V germline gene segment from the VH7(S107) gene family is introduced because VH domains of antibodies that express F(0.367), as we have shown previously (36), use this V gene segment in germline configuration (13, 16, 17). The CIS of the four V germline gene segments used by anti-DNA antibodies (31), such as BWDNA16, 2F2, BWDNA7, and VH283, which do not express F(0.367) is presented in Supplementary Table 1, revealing that characteristic peak is not at F(0.367).

In this report, the analysis was performed on 60 spontaneous anti-DNA VH domain sequences (31); of which, 20 are encoded by the V gene segments that express F(0.367). However, F(0.367) expression is limited, because only six antibody VH domains retained this characteristic (30%). Thus, we found that F(0.367) is expressed by IgG 74.c2 out of three individually analyzed VH domains of anti-DNA antibodies that use VH11 V gene segment or by IgG 17s-c2 out of nine analyzed anti-DNA antibodies that use S57(VH31) as well as IgG 17s.83, IgG 17s-c3, IgM 111.185, and IgM 165.27 out of eight analyzed VH domains encoded by MRL-DNA22 V gene segment. The CIS of the VH domains of these antibodies is shown in Figure 1B revealing a dominant peak at F(0.367). It might be concluded that some anti-DNA antibodies encoded by these V gene segments have lost F(0.367) as the result of somatic mutations.

In Figure 1C, CIS of VH domains for 54 anti-DNA VH domains is shown which, in an individual spectrum, does not express F(0.367) and thus do not encode epitope specificity for phosphate groups of dsDNA backbone. It should be emphasized that a peak at F(0.023) with dominant S/N value is detected for the V gene segments (Figure 1A), whereas it is a unique peak in the CIS (Figure 1C) obtained for anti-DNA VH domains, whose individual sequences do not express F(0.367) a feature relevant for the specificity here analyzed. An analysis of antibodies reactive with ssDNA, Z-DNA, and chromatin further confirms the connection between F(0.367) expression and antibody specificity for the phosphate group of B DNA backbone as shown in Supplementary Figures 1B–D.

It is of interest to note that comparison of the V gene segments and VH domain contribution to S/N value for the peak at F(0.367) revealed an insignificant CDR3 region contribution (Table 1).

**Table 1:** Contribution of antibody VH domains and corresponding V gene segments to S/N_F(0.367)_.

**Antibodies**	**Antibody isotype**	**V germline gene segment used**	**VH gene family**	**S/N**_**F(0.367)**_	
				**Domains**	**V gene segments**
T15	IgG	T15(V1)	VH7 (S107)^*^	3.727	3.544
U4	IgG	T15(V1)	VH7 (S107)^*^	3.950	3.821
74.c2	IgG	VH11	VH7 (S107)^*^	5.622	5.304
17s-c2	IgG	S57(VH31)	VH1 (J558)^*^	4.148	4.008
17s.83	IgG	DNA22	VH1 (J558)^*^	3.789	3.383
17s-c3	IgG	DNA22	VH1 (J558)^*^	4.434	3.944
111.185	IgM	DNA22	VH1 (J558)^*^	3.963	3.623
165.27	IgM	DNA22	VH1 (J558)^*^	3.731	3.259

* *Old nomenclature for VH gene families is given in parenthesis*.

We further made an attempt to determine peptide position in the V gene segment sequences mostly contributing to the F(0.367) expression. The data obtained showed that for T15(V1), VH11(VH7), S57[VH31(VH1)], and MRL-DNA22(VH1) V germline gene segments, these peptides involve residues at positions 35–66, 36–67, 46–65, and 46–77, respectively. The most important finding is that selected peptides include CDR2 regions that are abundant in basic residues (Table 2), indicating an CDR2 role in both F(0.367) expression and interaction with an antigenic determinant shared by the PC hapten and dsDNA.

**Table 2:** Sequence alignment for CDR2 regions of the V gene segments.

**V gene segments**	**Germline configuration**	**Antibody**	**CDR2 region amino acid sequence**																		
						abc	
MRL-DNA22	+		N	I	Y	P			G	S	S	S	T	**N**	Y	**N**	E	**K**	F	**K**	S
MRL-DNA22		111.185	–	–	–	–			–	–	–	–	–	–	–	–	–	–	–	–	–
MRL-DNA22		165.27	–	–	–	–			–	–	–	–	–	–	–	–	–	–	–	–	–
MRL-DNA22		17s.83	N	–	–	–			–	–	I	I	–	**H**	F	**N**	–	**K**	–	**K**	**N**
MRL-DNA22		17s-c3	E	–	–	–			**R**	–	G	**N**	I	Y	Y	**N**	–	**K**	–	**K**	G
						abc	
S57(VH31)	+		W	I	Y	S			G	S	G	**N**	T	**K**	Y	**N**	E	**K**	F	**K**	D
S57(VH31)		17s-c2	–	–	–	P			–	–	–	**N**	–	**K**	–	**N**	–	**K**	–	**K**	–
VH11	+		L	I	**R**	**N**	**K**	A	**N**	G	Y	T	T	E	Y	S	A	S	V	**K**	G
VH11		74-c2	–	–	**R**	**N**	**K**	–	**N**	**D**	–	–	–	–	–	–	–	–	–	**K**	–
						a	b	c
T15(V1)	+		**A**	**S**	**R**	**N**	**K**	**A**	**N**	**D**	**Y**	**T**	**T**	**E**	**Y**	**S**	**A**	**S**	**V**	**K**	**G**
T15(V1)		T15	–	–	**R**	**N**	**K**	–	**N**	–	–	–	–	–	–	–	–	–	–	**K**	–
T15(V1)		U4	–	–	**R**	**N**	**K**	–	**N**	–	–	–	–	–	–	–	–	–	–	**K**	–
VH1210.7	+		Y	I	S	Y	S	G	S	T	Y	Y	**N**	P	S	L	**K**	S			
VH1210.7		E7	–	–	–	–	–	–	–	–	–	–	–	–	–	–	–	–			
VH101	+		V	I	W	S	G	G	S	T	D	Y	**N**	A	A	F	I	S			
VH101		D23	–	–	–	–	–	–	–	–	–	–	–	–	–	–	–	–			

*The CDR2 region sequences (aa 50-65) for V germline genes and epitope specific anti-DNA antibodies shown in the single letter code were taken from Tillman et al. (31). Origin of these V germline gene sequences are: S57(VH31) (30), MRL-DNA22 (44), VH11 (45). Sequences for T15(V1), as well as E7 and D23 antibodies are taken from Diamond and Scharff (13), Crews et al. (16), Rudikoff et al. (17), and Baccala et al. (46) respectively. Within the compared antibody V gene segments dashes indicate identity with the reference germline gene sequences, while basic residues are shown in bold. Numbering is according to Kabat et al. (47)*.

The data obtained from the VH domains analysis of two preimmune natural polyreactive autoantibodies, E7 and D23 (46), which react with antigens such as DNA, myosin, actin, tubulin, spectrin, and trinitrophenol, revealed that F(0.367) was not expressed (Figure 1D), meaning that epitope specificity of these antibodies differs from dsDNA-reactive anti-DNA antibodies here analyzed. The CDR2 regions of these autoantibodies are in germline configuration and with a reduced number of basic residues.

## Discussion

Previously, using ISM for protein sequence analysis (37), we showed that antibody VH domains of T15 PC binding antibody and U4 dsDNA binding antibody encode information determining sequence specificity represented with characteristic frequency F(0.367), in short F(0.37) (36). We also showed that this frequency is dependent on the type of residues in the CDR2 region and insensitive to a residue substitution in CDR1 (36) of the T15 U4 mutant (13). In this report, we extend these findings by showing that F(0.367) is not only expressed by VH domains of T15 and some spontaneous anti-DNA antibodies from autoimmune mice but is found to be also intrinsic for the V germline gene segments used by these antibodies.

It has been shown that anti-PC binding antibody VH encoded by T15(V1) V gene segment of the VH7(S107) germline gene family (13, 16, 17) form strong interactions between the PC phosphate group and charged residues in the CDR2 region, such as Arg-52 and Lys-54, whereas CDR1 region Glu-35 is involved in choline binding (17, 18). Therefore, F(0.367) expressed by antibodies such as T15, T15 somatic mutant U4, and some anti-DNA antibodies may characterize epitope specificity, that is, specificity for phosphate groups present on different antigens such as PC hapten and dsDNA. Furthermore, the data presented showed that expression of the S/N_F(0.367)_ mostly depends on antibody V gene segments, and thus, a contribution of the CDR3 regions is insignificant (Table 1). It should be emphasized that IgG V gene segments of anti-DNA antibodies expressing F(0.367) can be close to germline configuration such as antibodies 74.c2 and 17s.83 encoded by the V gene segment VH11 of the VH7 (S107) gene family and MRL-DNA22 of the gene family VH1 (J558), respectively (31), suggesting that some mutations are tolerable as they do not affect the specificity delineated by the F(0.367). However, they differed in CDR3 regions (31), and their contribution to F(0.367) expression is insignificant (Table 1), whereas IgM 111.185 (MRL-DNA22) anti-DNA antibody (31) retains V gene segment in germline configuration. The data presented may be in accord with the idea that V germline gene segments prone to bind a dsDNA epitope should be less dependent on CDR3 regions (48).

Anti-dsDNA antibodies derived from autoimmune mouse models revealed that they have undergone somatic mutations suggesting their role in achievement of the corresponding conformation. Thus, an important finding obtained from sequence analysis showed the presence of basic amino acids Arg, Lys, and His and, perhaps, the uncharged Asn in CDRs (28–31), whereas Arg in the CDR3 has an important contribution in DNA specificity for DNA-histone complexes (32–34). However, cationic amino acids were not necessary for immune deposit formation (49). Thus, another goal of this study was to examine the role of the CDR2 regions in F(0.367) expression and in particular the content of basic residues in the CDR2 regions of V germline genes used by anti-DNA antibodies. The data obtained showed that peptides within sequences mostly contributing to F(0.367) expression cover residues at position 35–66 for T15(V1), 36–67 for VH11, 46–65 for S57(VH31), and 46–77 for MRL-DNA22. It should be emphasized that these peptide sequences include the CDR2 region enriched in basic residues of T15(V1), VH11, S57(VH31), and MRL-DNA22 germline genes and are also present in CDR2 regions of antibody VH domains (Table 2). It can be seen that type and positions of the basic residues for CDR2 of the T15(V1) and VH11 V germline gene segments and anti-DNA antibody V gene segments are the same and S57(VH31) differs slightly. The MRL-DNA22 anti-DNA IgM isotypes are close to germline configuration, whereas IgG differs in the type and position of basic residues (Table 2). The CDR2 regions of natural polyreactive autoantibodies are in germline configuration and have one or two basic residues, confirming that both the number and position in CDR2 regions are important for antibody epitope specificity.

The findings presented indicate that antibody specificity for an antigenic determinant (epitope) in the context of different antigens might be identified by the ISM approach (37).

The method applied made a possible correlation between primary antibody structure and specificity delineated by a characteristic frequency.

The main conclusion is that antibody VH domain sequences can encode ability expressed as characteristic frequency, to interact with non-protein structures of various molecules after achievement of the corresponding conformation by somatic mutations.

## Author contributions

TS-R and RM developed the study design, analyzed the data and wrote the paper. HK and VJ revised the paper.

### Conflict of interest statement

The authors declare that the research was conducted in the absence of any commercial or financial relationships that could be construed as a potential conflict of interest.

## Abbreviations

CDR: Hypervariable region
CIS: Cross-spectral analysis
EIIP: Electron-ion interaction potential
ISM: Informational spectrum method
IS: Informational spectrum
RRM: Resonant recognition model
S/N: Signal-to-noise ratio
VH: Variable heavy chain.
