# Supplementary material for: Antibody Epitope Specificity for dsDNA Phosphate Backbone Is an Intrinsic Property of the Heavy Chain Variable Germline Gene Segment Used
Source: Front Immunol. 2018 Oct 18;9:2378. doi: 10.3389/fimmu.2018.02378 (PMC6200867; doi:10.3389/fimmu.2018.02378)
Supplement: Supplementary file 1 [file Data_Sheet_1.docx]

**Supplementary Information**

Veljkovic et al (1), in Figure 1 of their article gave details of Informational Spectrum Method (ISM) employed here. ISM consists of several major steps including:

“(a) The alphabetic presentation of primary structures of p53 and MDM2 molecules. (b) The numerical presentation of primary structures of op53 and MDM2 molecules. (c) The spectral presentation of primary structures of p53 and MDM2 molecules. (d) cross-spectrum of p53 and MDM2 molecules. The abscissa represents the frequencies from the Fourier transform of the sequence of EIIP. The lowest frequency is 0.0 and the highest is 0.5. The ordinate represents amplitudes, in arbitrary units, corresponding to each frequency component in the informational spectrum (IS), normalized on the maximal amplitude value.” (Veljkovic et al.)

**Figure 1.** ISM analysis of the V germline genes and VH domains of anti-DNA antibodies of different epitope specificity and 3H9 chromatin reactive autoantibody.

CIS for V gene segments used by spontaneous anti-DNA antibodies (2) with references of sequence origin, which do not display F(0.367) such as S57(VH31) (3), MRL-DNA22 (4) and VH11 (5) (A). CIS of 17p.101, 17s.2, 17p.73, 17s.128, 165.45, 163.42 and 202.9 antibodies reactive with ssDNA DNA (2) (B), CIS of Z44 and Z22 antibodies which use V gene segments from VH10 (DNA4) reactive with Z-DNA a left-handed spiral (6). (C) IS of the 3H9 chromatin reactive antibody (7) (D). The abscissa represents the frequencies from the Fourier transform of the sequence of EIIP. The lowest frequency is 0.0 and the highest is 0.5. The ordinate represents the signal to noise ratio (S/N) corresponding to each frequency component in the informational spectrum (IS).

**Table 1.** Translated amino acid sequences for V germline gene segments and antibody VH domains (one-letter code). The sequences are grouped according to the VH family from which each VH was derived. An old nomenclature for each family is given in parentheses. Amino acid numbering is according to Kabat, et al. 1987: CDR1(30-35aa); CDR2 (50-65aa); CDR3(95-102aa).

**V germline gene segments**

**VH1 (J558) family**

1 . **BWDNA I 6**

EIQLQQSGAELVKPGASVKISCKASGYSFTGYNMNWVKQSHGKSLEWIGNINPYYGSTSYNQKFKGKATLTVDKSSSTAYMQLNSLTSEDSAVYYCAR

2. **2F2**

QVQLQQSGPELVKPGAVKISCKASGYAFSSSWMNWVKQRPGKGLEWIGRIYPGDGDTNYNGKFKDKATLTADKSSSTAYMQLSSLTSEDSAVYFCAR

3. **DNA22**

QVQLQQPGAELVKPGASVKLSCKASGYTFTSYWINWVKQRPGQGLEWIGNIYPGSSSTNYNEKFKSKATLTVDTSSSTAYMQLSSLTSDDSAVYYCAR

4. **BWDNA 7**

VKMSCKASGYTFTSYVMKWVKQKPGQGLEWIGYNDGTNYNEFKGKATLSSDKSSSTAYMELSSLTSEDSAVYYCAR

5. **S57 (Vh31)**

KPGASVKISCKASGYTFTDYYINWVKQRPGQGLEWIGWIYSGSGNTKYNEKFKDKATLTVDTSSSTAYMQLSSLTSEDSAVYFCAR

**VH5 (VH7183) family**

6. **VH283**

EVMLVESGGGLVKPGGSLKLSCAASGFTFSSYTMSWVRQTPEKRLEWVATISSGGGNTYYPDSVKGRFTISRDNAKNNVYLQMSSLRSEDTALYYCAR

**VH7 (S107) family**

7. **VH11**

EVKLVESGGGLVQPGGSLSLSCAASGFTSTDYYMSWVRQPPGKALEWLALIRNKANGYTTEYSASVKGRFTYSRDNSQSILYLQMNALRAEDSATYYCARD

**VH domains of spontaneously produced anti-DNA antibodies**

**VH7 (S107) family**

1. **163-c2**

EVNLVESGGGLVQPGGSLSLSCAASGFTFTDYYMNWVRQPPGKALEWLALIRNKANGYTTEYSASVKGRFTISRDNSQSILYLQMNVLRTEDSATYYCARDDPYGRTRSYTMDY

2. **74-c2**

EVKLVESGGGLVQPGGSLSLSCAASGFTFTDYYMSWVRQPPGKALEWLALIRKKANDYTTEYSASVKGRFTISRDDSQSILYLQMNALRAEDSATYYCARDKGRYGAWFAY

3. **111.33**

EVKLVESGGGLVQPGGSLSLSCAASGFTFTYYYMSWVRQPPGKALECLALIRNKANGYTTEYSASVKGRFTLSRDNSQSILYLQMNALRAEDSATYYCTRAASYGSRGWYFDV

**VH1 (J558) family**

4. **165.14**

QQSGAELVKPGASVKISCKASGYSFTGYNMNWVKQSHGKSLEWIGNINPYYGSTSYNQKFKGKATLTVDKSSSTAYMQLNSLTSEDSAVYYCARRYYGREGYYFDYW

5. **17s.128**

EIQLQQSGAELVKPGSVKISCKASGYSFTGYNMNWVKLSHGKSLEWIGNINPYYITTSYNQKFKGKATLTVDKSSSTAYMQLNSLTSEDSAVYYCARALRQGYYFDYW

6. **17s-c1**

EVQLQQSGPELVKPGASVKMSCKASGYTFTDYYMNWVKQSHGKSLEWIGRINPSNGGTSYNQKFKGKATLTVDKSLSTAYMQLNSLTSEDSAVYYCAREDYYGSSYFDYW

7. **165.60**

EVQLQQSGPELVKPGASVKMSCKASGYTFTDYYMNWVKQSHGKSLEWIGRVNPSNGGTSYNQKFKGKATLTVDKSLSTAYMQLNSLTSEDSAVYYCARGETTVVGKGYYFDYW

8. **163.72**

EVQLQQSGPELVKPGDSVKMSCKASGYTFTDYYMDWVKQSHGKSLEWIGYIYPNNGGTSYNQKFKGKATLTVDKSSSTAYMELHSLTSEDSAVYYCARRGITTVYFDYW

9. **25.12m**

QVQLQQSDRELVKPGSVKISCKASGYTFTDYNMDWVKQSHGKSLEWIGYIYPNNGGTGYNQKFKSKATLTVDKSSSTAYMELHSLTSEDSAVYYCARGRYTYADYW

10. **10-c1**

EVQLQQSGPELVKPGASVKISCKASGYSFTGYYVHWVKQSPEKSLESLGEINPRTGGTTYNQKFKAKATLTVDKSSSTAYMQLKSLTSEDSAVYYCTREDRTGYFDYW

11. **17s.166**

EVQLQQSGPELVKPGSVKISCKASGYSFTGYYMHWVKQSPEKSLEWIGEINPSTGGITYNQKFKAKATLTVDKSSSTAYMQLKSLTSEDSAVYYCARGRYTYADYW

12. **17s-c4**

QVQLQQSGPELVKPGAVKISCKASGYEFSRSWMNWVKQGPGKGLEWIGWIYPGDGDIKYNGKFKGRATLTADKSSSTAYMHLSSLTSEGSAVYFCAKGGNYGGSFYALDYW

13. **17s.13**

QVQLQQSGPELVKPGAVKISCKTSGYAFSSSWMNWVKQRPGKGLEWIGWIPRDGDTNYNGKFKDKATLTADISSNTAYMQLSSLTSEDSTVYFCARSRGYYFGSSRFFDVW

14. **111.185**

QVQLQQPGAELVKPGASVKLSCKASGYTFTSYWINWVKQRPGQGLEWIGNIYPGSSSTNYNEKFKSKATLTVDTSSSTAYMQLSSLTSDDSAVYYCARDGYAYW

15. **165.27**

QVQLQQPGAELVKSVKLSCKASGYTFTSYWINWVKQRPGQGLEWIGNIYPGSSSTNYNEKFKSKATLTVDTSSSTAYMQLSSLTSDDSAVYYCARDGYAYW

16. **17s.83**

QVQLQQPGAELVKPGASVKLSCKASGYTFTSYWINWVMQRPGQGLEWIGNIYPGSIITHFNEKFKNKATLTVDTSSSTAYMQLSSLTSDDSAVYYCAGGYKAWFVY

17. **163.100**

QVQLQQPGAELVKPGASVKLSCKASGYTFTSYWMKWVKQRPGQGLEWIGEINPSNGGTNYNEKFKSKATLTVDKSSSTAYMQLSSLTSEDSAVYYCTIRLRWAWFAYW

18. **17s-c6**

QVQLQQPGAELVKPGASVKLSCKASGYTFTSYWMNWVKQRPGRGLEWIGRIHPSDSETHYNQKFKSKATLTVDKSSTTAYIQLSSLTSDDSAVYYCARRFAYW

19. **163.47**

QVQLQQPGAELVKPGASVKLSCKASGYTFTNYWMNWVKQRPGRGLEWIGRILPSDTETHYNQKFKNKATLTVDKSTSTAYIRLSSLTSEDSGIYYCARGIYWYFDVW

20. **165.49**

GAELVKPGASVKLSCKASGYTFTSYWINWVKQRPGQGLEWIGNIYPGSSSTNYNEKFKSKATLTVDTSSSTAYMQLSSLTSDDSAVYYCARRAWDWYFDVW

21. **17s-c3**

QVQLQQSGAELVARGASVKLSCRASGYTFTNYDINWVRQRAGQGLEWIGEIYPRSGNIYYNEKFKGKATLTADKSSSTTYMHLSGLTSGDSAVYYCAREGWEGGPYYFDYW

22. **111-c1**

EVQLQQSGPELVKPGASVKMSCKASGYTFTSYVMKWVKQKPGQGLEWIGYNDGTKYNEFKGKATLTSDKSSSTAYMELSSLTSEDSAVYYCARGGSGYDGFAYW

23. **111-c2**

EVQLQQSGPELVKPGASVKMSCKASGYTFTRYVMKWVKQKPGQGLEWIGYNDGTKYNEFKGKATLTSDKSSSTAYMELSSLTSEDSAVYYCARGTVIGDYYAMDYW

24. **17p.101**

EVQLQQSGPELVKPGVVKMSCKASGYTFTSYVMKWVKQKPGQGLEWIGYNDGTKYNEFKGKATLTSDKSSSTAYMELSSLTSEDSAVYYCASLIYFDYW

25. **202.80**

EVQLQQSGPELVKPGASVKMSCKASGYTFTSYVMKWVKQKPGQGLEWIGYNDGTKYNEFKGKATLTSDKSSSTAYMELSSLTSEDSAVYYCARRGYYGSSYAMDYW

26. **202.S38**

EVQLQQSGPELVKPGASVKMSCKASGYTFTSYVMKWVKQKPGQGLEWIGYNDGTKYNEFKGKATLTSDKSSSTAYMELSSLTSEDSAVYYCARGGRYDLYYAMDYW

27. **202.135**

EVQLQQSGPELVKPGASVKMSCKASGYTFTSYVMKWVKQKPGQGLEWIGYNDGTKYNEFKGKATLTSDKSSSTAYMELSSLTSEDSAVYYCARGYYGSSYSWFAYW

28. **202.61**

EVQLQQSGPELVKPGASVKMSCKASGYTFTSYVMKWVKQKPGQGLEWIGYNDGTKYNEFKGKATLTSDKSSSTAYMELSSLTSEDSAVYYCARLIYYYGSIGFAYW

29. **165. 3m**

MSCKASGYTFTSYVMKWVKQKPGQGLEWIGYNDGTKYNEFKGKATLTSDKSSSTAYMELSSLTSEDSAVYYCARDPPLRRLYYYAMDYW

30. **17s-c2**

QIQLQQSGPELVRPGASVKISCKASGYTFTDYYINWVKQRPGQGLEWIGWIYPGSGNTKYNEKFKGKATLTVDTSSSTAYMQLSSLTSEDSAVYFCARRGRSVYYFDY

31. **165.3**

QIQLQQSGPELVRPGASVKISCKASGYTFTDYYINWVKQRPGQGLEWIGWIYPGSGNTKYNEKFKGKATLTVDTSSSTAYMQLSSLTSEDSAVYFCARGDLLWLRRILDY

32. **74-c1**

QIRLQQSGPELVRPGSVKISCKASGYTFTDYYINWVKQRPGQGLEWIGWIYPGSGNTKYNEKFKGKATLTVDTSSSTAYMQLRSLTSEDSAVHFCAKEDWDGGFVYW

33. **202.105**

EVQLQQSGPELVKPGASVKISCKASGYTFTDYYMHWVKQKPGQGLEWIGEIYPGSGNTYYNEKFKGKASLTADKSSSTAYMQLSSLTSEDSAVYFCARRYYRRSYAMDY

34. **111.68**

QVQLQQSGPELVKPGASVKLSCKASGYTFTDYTIHWVKQSPGQGLEWIGWIYPGSGNTKYNDKFKGKATMTADKSSSTAYMQLSSLTSEDSAVYFCARGVARGSAMDYW

35. **165.45**

QVQLQQSGPELVKPGASVKLSCKASGYTFTDYTIWVKQSPGQGLEWIGWIYPGSGNTKYNDKFKGKATMTADKSSRTAYMQLSSLTSEDSAVYFCAREAYWYFDVW

36. **17s-c5**

QVQLQQSEAELARPGASVKMSCKASGYTFTRYWMHWVKQRPGQALEWIGAIYPGNSDTNQKFKGKAKLTAVTSASTAYMELSSLASEDSAVYYCARSRYRGYSMDY

37. **165.5**

KMSCKASGYTFTNYWMHWVKQRPGQGLEWIGAIYPGNSDTSQKFKGKAKLTAVTSASTAYMELSSLTNEDSAVYYCTREGCYYWYFDVW

38. **202.54**

QVQLQQSGAELAKPGASVKMSCKASGYSFTRYWMHWVKQRPGQGLEWIGYINPSSGYTEQKFKDKATLTADKSSSTAYMQLSSLTSEDSAVYYCASLPWYFDVW

**VH5 (7183) family**

39. **202.33**

ESGGALVKPGGSLKLSCAASGFTFSSYGMSWVRQTPEKRLEWVATISGGGGNTYYPDTVKGRFTISRDNAKNTLYLQMSSLRSEDTALYYCARSRWLLRVGYWYFDVW

40. **163-c1**

EVKLVESGGALVKPGGSLKLSCAASGFTFSSYAMSWVRQTPAKRLEWVAYISGGGSYTYYPDSVKGRFTISRDNAKNTLYLQMSSLRSEDTAMYYCARHYYGSRTYYFDYW

41. **83-c1**

EVKLMESGGGLVKPGGSLKLSCAASGFTFSSYVMSWVRQTPAKRLEWVAYISSGGDSTYYPDNMKGRFTISRDNAKNTLYLQMSSLRSEDTAMYYCARGGTRFAYW

42. **17s.5**

EVMLVESGGALVKPGGSLKLSCAASGFTFSDYAMSWVRQTPQKRLEWVATISSGGIYTYYPDGVQGRLTISRDNARNTLYLQMSSLRSEDTAMYYCARRGTTVYFDYW

43. **17s.93**

EVQLVESGGALVKPGGSLKLSCAASGFTFSNYAMSWVRQTPEKRLEWVATISSGGSYTYYPDSVKGRFTSRDNAKNTLYLQMSSLRSEDTALYYCARRGTTVYFDYW

44. **202.38m**

EVQLVESGGGLVKPGGSLKLSCAASGFTFSSYAMSWVRQTPEKRLEWVAAINSNGGSTYYPDTVKDRFTISRDNAKNTLYLQMSSLRSEDTALYYCARQGWDRAMDYW

45. **17s.2**

EVQLVESGGGLVKPGGSRKLSCAASGFTFSDFGIHWVRQAPEKGLEWVAYISRGSGTIYYADTVKGRFTISRDNDKNILFLQMTSLRSEDTAIYYCARNLGRRTYYFDY

46. **111.55**

EVQLVESGGGLVKPGGSRKLSCAASGFTFSDYGMHWVRQAPEKGLEWVAYISSGSFNIYYADTVKGRFTISRDNAKNTLFLQMTSLRSEDTAMYYCARNMATAWFVYW

47. **163.42**

GLVKPGGSRKLSCAASGFTFSDYGMHWVRQAPEKGLEWIAYISGGSNTIYYADTVKGRFTIFRDNAKNTLFLQMTSLRSEDTAMYYCARRYYGTFLFDYW

48. **163-c4**

GLVKPGGSRKLSCAASGFTFSDFGMHWVRQAPEKGLEWVAYISSGSSTIYYADTVKGRFTISRDNAKNTLFLQMTSLRSEDTAMYYCARKGLRRNYYAMDDW

49. **DNA13**

EVKLVESEGGLVQPGSSMKLSCTVFGFSFSDYYMAWVRQFPEKGLEWVAKINYDGSNTYYLDSLKGRFIISRDNAKNILYLQMSSLKSEDTATYFCSRVNYNGLRRSCFAD

50. **17s.130**

EVKLVESEGGLVQPGSSMKLSCTASGFTFSDYYMAWVRQVPEKGLEWVANINYDGSSTHYLDSLKGRFIISRDNGKNILYLQMSSLKSEDTATYYCTRDLKWLRRGYWYFDVW

51. **17p.73**

EVQLQQSGAELVRPGASVKLSCTASGFNIKDDYMHWVKQRPEQGLEWIGRIDPANGNTKYAPKFQDKATITADTSSNTAYLQLSSLTSEDTAVYYCARRDNYYAMDY

52. **111.67**

EVQLQQSGAELVRPGASVKLSCTASGFNIKDDYLHWVKQRPEQGLEWIGRIDPANGNTKYAPKFQDKATITADTSSNTAYLQLSSLTSEDTAVYYCARGSIWYFDVW

**VH2 (Q52)**

53. **165.33**

QVQMKESGPDLVQPSQTLSLTCTVSGFSLSSYGVHWFRKPPRKGLEWLGGIWSGGSIYYTALSSRLSVSRDISKSQVFFKMSSLQSEDTAVYHCARYYYGSPLNWYFDV

54. **165.41**

QVQMKESGPDLVQPSTLSLTCTVSGFSSSYGVHWFRKPPRKGLEWLGGIWSGGSIYYTALSSRLSVSRDISKSQVFFKMSSLQSEDTAVYHCARYDGYYYFDYW

55. **165.52**

PDLVQPSQTLSLTCTVSGFSLSSYGVHWFRKPPRKGLEWLGGIWSGGSIYYTALSSRLSVSRDISKSQVFFKMSSLQSEDTAVYHCARYHSTAPWWFDVW

56. **202.17**

QVQMQESGPDLVQPSQTLSLTCTVSGFSLSSYGVHWFRKPPRKGLEWLGGIWSGGSIYYTALSSRLSVSRDISKSQVFFKMSSLQSEDTAVYHCARYSDYYGSSYWYFDVW

57. **185-c1**

QVQLQESGPGLVAPSQSLSITCTVSGFSLTSYAISWVRQPPGKGLEWLGVIWTGGDTSYNSLSKRLSISKDNSKSQVFLKMNSLQTDDTARYYCARNTPLGRRYYFDYW

58. **17s.145**

QVQLKESGPVLVAPSQSLSITCTVSGFSLTSYGVHWVRQPPGKGLEWLGVIWAGGSTNYNSTLMSRLSISKDNSKSQVFLKMNSLQADDTAMFYCVKHKYYDISPFAFW

59. **202.9**

QVQLKESGPDLVQPSQTLSLTCTVSGFSLSSYGVHWVRQPPGKGLEWVGTMGWGDKKYYNSLKSRLSISRDTSKNQVFLKLSSLQTEDTAMYYCTYYYGSSYYAMDYW

60. **163-c3**

QVQLKQSGPGLVQPSQSLSITCTVSGFSLTNYGVHWVRQSPGKGLEWLGMIWSGGNTDYNALFIRLSISKDNSKSQVFFKMNSLQADDTATYFCARKGLRRAGAMDYW

**Natural polyreactive autoantibodies**

**V germline gene segments**

**VH3(36-60) family**

1**. VH1210.7**

EVQLQESGPSLVKPSQTLSLTCSVTGDSITSGYWNWIRKFPGNKLEYMGYISYSGSTYYNPSLKSRISITRDTSKNQYYLQLNSVTTEDTYCAR

**VH2 (Q52) family**

2. **VH101**

QVQVLKQSGPGLVQPSQSLSITCTVSGFSLTSYGVRWVRQSPGKGLEWLGVIWSGGSTDYNAAFISRLSISKDNSKSQVFFKMNSLQANDTAIYYCAR

**VH domains of natural polyreactive autoantibodies**

**VH3(VH 36-60) family**

1. **E7**

EVQLQESGPSLVKPSQTLSLTCSVTGDSITSGYWNWIRKFPGNKLEYMGYISYSGSTYYNPSLKSRISITRDTSKNQYYLQLNSVTTEDTYCARRPFYGKGAMDY

**VH2(Q52) family**

2. **D23**

QVQVLKQSGPGLVQPSQSLSITCTVSGFSLTSYGVRWVRQSPGKGLEWLGVIWSGGSTDYNAAFISRLSISKDNSKSQVFFKMNSLQANDTAIYYCAREKLRLRYFDY

**Anti-PC antibodies**

**V germline gene segments**

**VH7(S107)family**

**1. T15(V1)**

EVKLVESGGGLVQPGGSLRLSCATSGFTFSDFYMEWVRQPPGKRLEWIAASRNKANDYTTEYSASVKGRFIVSRDTSQSILYLQMNALRAEDTGIYYCARD

**VH domains of Anti-PC antibodies**

**VH7(S107)family**

1. **T15[T15(V1)]**

EVKLVESGGGLVQPGGSLRLSCATSGFTFSDFYMEWVRQPPGKRLEWIAASRNKANDYTTEYSASVKGRFIVSRDTSQSILYLQMNALRAEDTAIYYCARDYYGSSYWY

2. **U4[T15(V1)]**

EVKLVESGGGLVQPGGSLRLSCATSGFTFSDFYMAWVRQPPGKRLEWIAASRNKANDYTTEYSASVKGRFIVSRDTSQSILYLQMNALRAEDTAIYYCARDYYGSSYWY

**Anti-DNA reactive with Z-DNA**

**VH10(DNA4) family**

1. **Z22**

MLLGLKWVFFVVFYQGVHCKVQLVESGGGLVQPKGSLKLSCAASGFNFNTYAMNWVRQAPGKGLEWVARIRSKSNNYATYYADSMKDRFTISRDDSENMLYLQMINLKAEDTAMYYCVRQAYSNYGAMDYWGQGISVTVSS

2. **Z44**

MVLGLKWVFFVVFYQGVLCEVQLVESGGGLVQPKGSLKLSCAASGFTFNTYAMHWVRQAPGKGLEWVARIRSKSSNYATYYADSVKDRFTISRDDSQSMLYLQMNNLRTEDTAMYYCVREQLRYYYVMDYWGQGTSVTVSS

**Anti-DNA antibody reactive with chromatin**

**V germline gene segment**

**VH1(J558) family**

1. **VH133.16**

QVQLQQSEPGLVKPGGSVKISCKASGTAFSSSWMNWVKQRPGKGKEWIGRIYPGDGDTNYNGKFKGKATLTADKSSSTAYMQLSSLTSEDSAVYFCAR

**VH domain of anti-DNA antibody reactive with chromatin**

**VH1(J558) family**

1. **3H9**

QVQLQQSGPELVKPEASVKISCKASGYAFSSSWMNWVKQRPGKGLEWIGRIYPGDGDTTNNGKFKDKATLTADKSSSTAYMQLSSLTSEDSAVYFCARARSKYSYVMDY

**References**

1. Veljkovic N, Glisic S, Prljic J, Perovic V, Botta M, Veljkovic V. Discovery of new therapeutic targets by the informational spectrum method. Curr Protein Pep Sci (2008) 9: 493–506.

2. Tillman DM, Jou NT, Hill RJ, Marion TN. Both IgM and IgG anti-DNA antibodies are the products of clonally selective B cell stimulation in (NZB x NZW)F1 mice. J Exp Med (1992) 176: 761–779.

3. Shlomchik M, Mascelli M, Shan H, Radic MZ, Pisetsky D, Marshak-Rothstein A, et al. Anti- DNA antibodies from autoimmune mice arise by clonal expansion and somatic mutation. J Exp Med (1990) 171: 265–292.

4. Kofler, R, Strohal R, Balderas RS, Johnson ME, Noonan DJ, Duchosal MA, et al. Immunoglobulin k light chain variable region complex organization and immunoglobulin genesencoding anti-DNA autoantibodies in lupus mice. J Clin Invest (1988) 82: 852-860.

5. Clarke SH, Claflin JL, Rudikoff S. Polymorphisms in immunoglobulin heavy chains suggesting gene conversion. Proc. Natl Acad Sci US. (1982) 79: 3280-3284.

6. Brigido MM, Stollar BD.Two induced anti-Z-DNA monoclonal antibodies use VH gene segments related to those of anti-DNA autoantibodies. J Immunol (1991) 146: 2005-2009.

7. Shlomchik MJ, Aucoin AH, Pisetsky DS, Weigert MG. Structure and function of anti-DNA autoantibodies derived from a single autoimmune mouse. Proc Natl Acad Sci USA (1987) 84: 9150-9154.
